# Effects of Transmitters and Amyloid-Beta Peptide on Calcium Signals in Rat Cortical Astrocytes: Fura-2AM Measurements and Stochastic Model Simulations

**DOI:** 10.1371/journal.pone.0017914

**Published:** 2011-03-29

**Authors:** Eeva Toivari, Tiina Manninen, Amit K. Nahata, Tuula O. Jalonen, Marja-Leena Linne

**Affiliations:** 1 Department of Signal Processing, Tampere University of Technology, Tampere, Finland; 2 Kidney Care Center at DCI, Steubenville, Ohio, United States of America; 3 Department of Physiology and Neuroscience, School of Medicine, St. George's University, Grenada, West Indies; University of Maribor, Slovenia

## Abstract

**Background:**

To better understand the complex molecular level interactions seen in the pathogenesis of Alzheimer's disease, the results of the wet-lab and clinical studies can be complemented by mathematical models. Astrocytes are known to become reactive in Alzheimer's disease and their ionic equilibrium can be disturbed by interaction of the released and accumulated transmitters, such as serotonin, and peptides, including amyloid-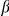 peptides (A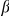). We have here studied the effects of small amounts of A
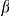
25–35 fragments on the transmitter-induced calcium signals in astrocytes by Fura-2AM fluorescence measurements and running simulations of the detected calcium signals.

**Methodology/Principal Findings:**

Intracellular calcium signals were measured in cultured rat cortical astrocytes following additions of serotonin and glutamate, or either of these transmitters together with A
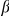
25–35. A
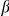
25–35 increased the number of astrocytes responding to glutamate and exceedingly increased the magnitude of the serotonin-induced calcium signals. In addition to A
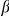
25–35-induced effects, the contribution of intracellular calcium stores to calcium signaling was tested. When using higher stimulus frequency, the subsequent calcium peaks after the initial peak were of lower amplitude. This may indicate inadequate filling of the intracellular calcium stores between the stimuli. In order to reproduce the experimental findings, a stochastic computational model was introduced. The model takes into account the major mechanisms known to be involved in calcium signaling in astrocytes. Model simulations confirm the principal experimental findings and show the variability typical for experimental measurements.

**Conclusions/Significance:**

Nanomolar A
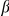
25–35 alone does not cause persistent change in the basal level of calcium in astrocytes. However, even small amounts of A
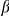
25–35, together with transmitters, can have substantial synergistic effects on intracellular calcium signals. Computational modeling further helps in understanding the mechanisms associated with intracellular calcium oscillations. Modeling the mechanisms is important, as astrocytes have an essential role in regulating the neuronal microenvironment of the central nervous system.

## Introduction

Alzheimer's disease (AD) is a progressive and irreversible neurodegenerative disorder that leads to cognitive impairment and emotional disturbances. Symptoms result from the degeneration of brain tissue, seen as shrinkage of certain brain regions, which are involved in cognitive processes, learning, and memory formation (reviewed in [1]). In addition to brain shrinkage, AD patients suffer from accumulation of amyloid-beta (A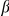) containing neuritic plaques and neurofibrillary tangles (tau protein in neuronal somata), which are considered as hallmarks of AD. Though the pathological changes in the brain can be detected using MRI and PET imaging techniques, the exact molecular mechanisms leading to the severe symptoms are not yet known. Early diagnosis together with a possibility of specific targeted treatment would provide the patients with more years of quality life.

Amyloid plaques containing aggregated A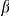 fragments have been shown to disturb the homeostasis of intracellular calcium ions (Ca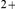) and contribute to the altered Ca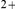 signaling in the brain cells [1]. The plaques typically consist of 39–42 amino acid A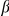 fragments, and the plasma ratio of 42 and 40 amino acids long fragments (A
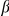
42/A
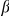
40) is suggested of being useful for identifying the risk of developing mild cognitive impairment and AD [2], [3]. Based on the classification of amino acids by Branden and Tooze [4], 25 amino acids out of the total 42 have hydrophobic side chains in A
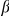
42. Therefore, A
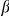
42 tends to aggregate easier than the shorter A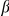 fragments. A
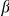
42 and the shorter 11 amino acids long synthetic derivative (A
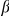
25–35) are both fragments which are widely used in Alzheimer's disease research (see recent studies [5]–[10]) with specifically A
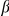
25–35 having Ca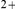-mediated neurotoxic properties [11], [12].

So far the *in vitro* studies of the effects of A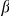 peptide on the cellular Ca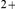 responses have failed to give any definite answers to the mechanisms involved. Together with the longer fragments, A
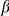
25–35 has been shown to depress hippocampal long-term potentiation [13] and to potentiate the long-term depression [14], both of which depend on the increases in intracellular Ca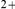 concentration in neurons. A
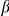
25–35 has been shown to induce transient changes in intracellular Ca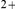 concentration in astrocytes [15], [16]. These effects may be important in explaining the loss of new memory formation and learning seen in AD. The detailed mechanisms behind the A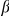-induced neuronal and glial Ca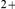 fluctuations, as well as the changes triggered by these, require further studies.

One of the central functions of astrocytes is gliotransmitter/neurotransmitter release and uptake in the neuronal synaptic cleft of the tripartite synapse [17] together with more complex regulation of the neuronal microenvironment [18]–[23]. Astrocytes thus have a vital role in the synaptic information processing and in the metabolism of the central nervous system. Astrocytes release transmitters and have receptors and transporters for different neurotransmitters in their plasma membranes, such as for serotonin (5-hydroxytryptamine; 5-HT), ATP, and glutamate [17], [24]. Astrocytes, as well as other glial cells, use both spontaneous and stimulated variations of the Ca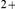 concentration for intra- and intercellular signaling [25], [26]. Previous electrophysiological and Ca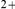 imaging studies have shown how already micromolar concentration of 5-HT cause transient release of Ca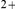 from intracellular stores followed by prolonged transmembrane inward Ca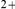 flow [17], [27]. We here have used rat cortical astrocytes, similarly to our earlier studies on A
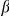
25–35 and A
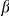
1–40 [16], to study the special effects of A
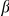
25–35 to Ca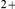 signals when added together with transmitters. We now show that A
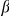
25–35 increases the initial peak of Ca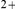 release when added together with 5-HT, compared to the effects of 5-HT alone.

Despite the rapid advancements in computing technology, it is currently not possible to model mathematically the biological systems of realistic complexity over interesting time scales by only using the molecular dynamic approach [28]. Typically, the details of the state of the system (such as the position, orientation, and momentum of individual particles) are excluded in the modeling of whole-cell level phenomena. Here, we describe a model of astrocyte Ca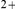 signals as a macroscopic flow of Ca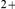 ions rather than as a model of each individual Ca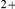 channel in the membranes. In the case of AD, abnormal Ca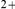 signals could be among the first hallmarks of disturbed brain function (the correlation between Ca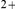 and A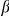 is reviewed in [29]). A computational model which closely mimics the experimentally measured Ca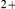 signals in rat cortical astrocytes helps in understanding the interaction of the various components of Ca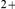 dynamics in healthy cells versus the cells with dysfunctional metabolism.

## Methods

### Experimental methods and data

#### Ethics Statement

Confluent primary astrocyte cultures were prepared from cortices of newborn Sprague-Dawley rat pups as previously described [30], with minor modifications. Pups were killed by decapitation according to the procedure conforming to the Public Health Service Policy on Humane Care and Use of Laboratory Animals and approved by the Albany Medical College Institutional Animal Care and Use Committee for Dr. H.K. Kimelberg, Protocol ID 006038 entitled “Neurotransmitter receptors and ion channels on astrocytes”.

#### Cell culture

Primary astrocyte cultures were prepared from new-born Sprague-Dawley rat pups. In brief, the cerebral hemispheres were removed, freed from the meninges and mechanically dissociated using Dispase (Sigma, St. Louis, MO, USA) into culture medium (Eagle's Medium with Earle's salts, Gibco, U.K.) supplemented with 10% heat-inactivated horse serum (HS, Gibco, U.K.), 25 mM sodium bicarbonate and antibiotics (penicillin and streptomycin). Deoxyribonuclease I (Sigma, St. Louis, MO, USA) was added to prevent cell clumping during the second extraction. Cells were grown on coverslips in culture dishes and kept at 37°C in an air-ventilated humidified incubator containing 5% CO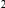. The medium was first changed after one day and subsequently twice a week. About 95% of the cells routinely stained positively for glial fibrillary acidic protein (GFAP+), with polyclonal rabbit anti-cow GFAP used as the primary antibody and either rhodamine or fluorescein conjugated gamma & light chain goat anti-rabbit IgGs as secondary antibody. The studies were performed on cells kept for 1 to 4 weeks in culture.

#### Calcium imaging

Fura-2-acetoxymethyl ester (Fura-2AM) is a membrane penetrating derivative of the radiometric Ca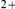 indicator Fura-2 used to measure intracellular Ca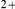 concentrations by fluorescence. Inside the cell, the acetoxymethyl groups in Fura-2AM are removed by cellular esterases resulting to generate Fura-2, the pentacarboxylate Ca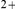 indicator. The ratio of the emissions at 340 and 380 nm wavelengths is directly correlated to the amount of intracellular Ca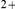 concentration (as presented in [17]). Calcium imaging of Fura-2AM-loaded astrocytes was performed using a monochromator based spectrophotofluorimetric system (Model RF D-4010 Deltascan, PTI, USA, PC computer and software together with Nikon Diaphot microscope) with dual excitation at the 340 and 380 nm wavelengths, bandpass of 2 nm and the fluorescence emission measurements at 510 nm wavelength. Astrocytes on the coverslips were loaded for 30 minutes at 37°C in a HEPES-buffered Ringer's solution containing 4 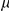
M Fura-2AM (Molecular Probes, Inc., Eugine, OR). The coverslips were then rinsed and placed in a Sykes-Moore Culture Chamber (Bellco Biotech, Vineland, NJ, USA). Experiments were made at room temperature. For determining the effects of transmitters, a stable baseline for intracellular Ca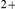 concentration was first obtained, after which the solution was replaced normally for 30 s by a buffer solution containing 5-HT or glutamate. After this the transmitter was rinsed away by several successive applications of a fresh buffer solution. Results are shown as a ratio of the emissions obtained by the two wavelengths of 340 nm and 380 nm.

#### Solutions

Solutions of the following composition were used and the chemicals were obtained from Sigma, St.Louis, MO. USA, if not otherwise stated.

1. Ca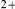-imaging Ringer (mM): NaCl 122, KCl 3.3, MgSO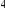 0.4, CaCl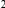 1.3, KH
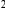
PO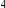 1.2, HEPES 25, Glucose 10, and sucrose to balance the osmolarity to 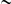
320 mOsmol., pH 7.35–7.4.
2. A
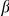
25–35 peptide (RBI, Natick, MA, USA) was first dissolved in water and then diluted in buffer solution. 10 nM, 200 nM and 1 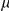
M final concentration of the peptide was used either acutely or with incubation.
3. 1 or 10 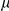
M final concentration of 5-HT HCl (for references see [17], [27]), or 50 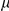
M L-glutamate were added in the recording chamber.

**Figure 1:**
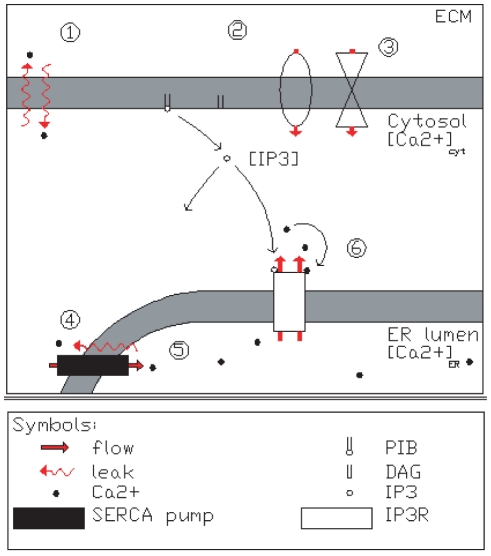
Graphical illustration of the events affecting the astrocytic Ca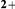 concentration. For the explanation of model components 1–6, see text. In short, according to general knowledge, the primary messengers pass their effects via their respective receptors and G proteins on the plasma membrane. Once activated, the G protein activates the membrane-bound phospholipase C (PLC). Furthermore, active PLC propagates its signal by cleaving a lipid molecule phosphatidyl-inositol 4,5-bisphosphate (PIB) that is in attendance in small quantities in the inner half of the plasma membrane lipid bilayer. By disassociating the sugar-phosphate head of the PIB, PLC generates two separate second messenger molecules; inositol 1,4,5-trisphosphate (IP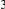) and diacylglyserol (DAG). While DAG remains embedded in the plasma membrane, hydrophilic IP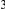 diffuses into the cytosol, binds to its receptor (IP
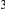
R) on ER causing Ca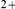 liberation to cytosol.

**Figure 2:**
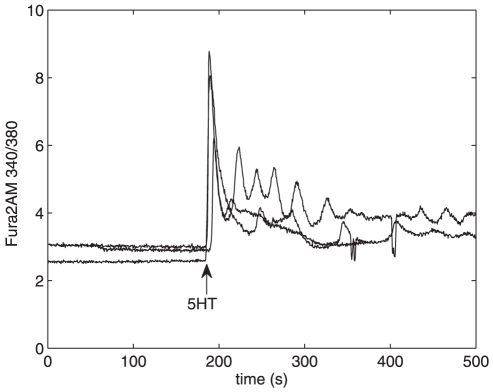
Changes in cytosolic Ca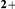 concentration induced by 5-HT and A
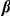
25–35. Changes in cytosolic Ca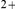 concentration is measured with Fura-2AM. Similar experimental conditions (incubation for 48 hours with 200 nM A
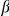
25–35 and an acute addition of 10 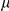
M 5-HT at t = 180 s) were used for all curves. Although there is variability in the measured Ca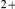 signals, the trend in the curves is the same.

The obtained experimental data together with the known components affecting cellular Ca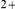 concentration (presented in Figure 1) were used to design the type of the computational model. Because of the variability of detected Ca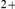 levels (see Figure 2), stochasticity was introduced into the computational model which was validated by the data obtained from Fura-2AM measurements.

### Stochastic model for Ca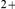 signals

Computational modeling, in general, means mathematical description of the functional properties of the system components and the analysis of the model predictions. One of the challenges in computational modeling is the lack of precise experimental data for model components. In other words, a specific experimental data set with proper statistics is needed for selecting relevant range of values for model parameters. Validation of the model is typically done by comparing the predicted output of the model with the experimental data. To ensure the relevant parameter values, a computational model for Ca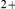 signaling in astrocytes by Di Garbo et al. [31] was taken as a reference model. The model takes into account the physiological phenomena known to be the major contributors in the intracellular Ca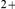 oscillations. In summary, it describes the Ca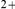 concentration in cytosol as a six-component system (a graphical illustration in Figure 1). Namely, 1) Ca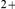 leak from/to extracellular matrix (ECM), 2) capacitive Ca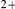 entry (CCE) from ECM, 3) Ca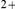 entry via ionotropic receptors, 4) Ca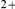 leak from intracellular stores, such as endoplasmic reticulum (ER), 5) storage of Ca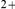 to ER via sarco(endo)plasmic Ca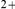 ATPase (SERCA) pumps, and 6) Ca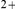 release from ER mediated by inositol 1,4,5-trisphosphate (IP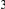). The reference model carefully addresses a widely accepted mechanism for astrocytic Ca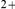 increases via the canonical G protein/phospholipase C (PLC)/IP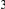 pathway [32] where the IP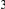 released into the cytosol binds to its receptor (IP
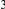
R) on ER, the Ca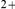 channels open, and Ca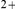 ions inside the ER are liberated to cytosol causing a sharp rise in the cytosolic concentration of free Ca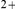 which is normally kept very low (see also [33], [34]). The parameters used for both the reference model (deterministic; Di Garbo et al. [31]) and the here developed stochastic model are presented in Table 1.

**Table 1:** Model parameters and used parameter values of the computational model.

Symbol	Value	Explanation
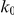	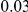 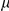 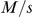	Rate of Ca 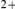 leak across the plasma membrane
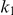	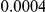 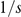	Rate of Ca 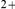 leak from the ER
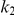	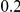 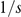	Rate of Ca 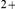 release through IP 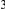 receptor
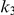	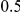 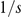	Rate constant of SERCA pump
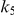	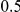 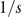	Rate of Ca 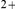 extrusion from plasma membrane
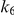	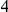 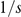	Rate constant of IP 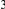 receptor inactivation
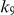	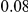 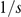	Rate constant of IP 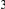 degradation
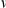 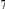	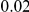 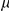 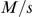	Rate constant of PLC 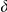
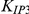	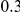 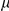 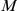	Half saturation constant for IP 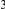 activation of the
		corresponding receptor
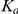	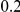 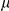 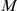	Half saturation constant for Ca 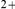 activation of the IP 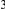 receptor
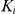	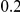 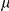 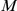	Half saturation constant for Ca 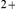 inhibition of the IP 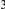 receptor
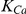	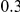 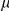 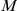	Half saturation constant for Ca 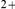 activation of PLC 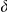
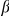	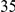	Ratio of the effective volumes for Ca 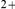 of cytoplasm and ER
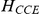	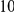 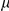 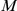	Half inactivation constant for CCE influx
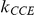	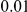 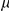 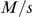	Maximal rate constant for CCE influx
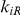	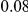 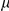 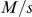	Maximal rate of stimuli-evoked ionotropic Ca 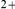 flux
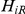	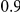 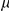 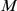	Half saturation constant for stimuli-evoked ionotropic Ca 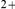
		influx amplitude
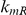	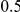 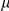 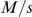	Maximal rate of IP 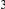 production mediated by the metabotropic
		receptor
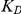	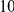 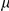 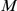	Dissociation constant for the binding of ligand/metabotropic receptor
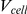	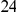 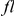	Volume of the cell
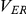	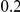 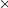 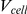	Volume of the ER
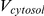	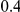 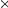 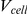	Volume of the cytosol

Model parameters and parameter values used both in the deterministic reference model and in the stochastic model introduced in this study, excluding the last three volumes which were only used in the stochastic model. More information and references for the used values can also be found from [31]. Used abbreviations: capacitive Ca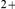 entry (CCE), endoplasmic reticulum (ER), inositol 1,4,5-trisphosphate (IP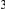), sarco(endo)plasmic Ca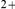 ATPase (SERCA), and phospholipase C (PLC).

The kinetics of biological processes are typically stochastic, i.e. random, in nature [28]. Therefore, the cellular functions cannot be properly understood with purely deterministic models (see, e.g., [35]–[37]) and both the intrinsic and extrinsic stochastic phenomena need to be accounted for *in silico* models. Intrinsic stochasticity is caused by the dynamics of the system from the random timing of individual reaction events. The importance of intrinsic stochasticity becomes obvious in systems with low numbers of molecules. However, stochasticity included in the model may not always be able to explain the large diversity observed in experimental measurements (as shown in [38]). The low numbers make individual reaction events, which change molecular numbers by one or two, more significant. At the same time the extrinsic stochasticity is caused by the system interacting with other stochastic systems in the cell or its environment. Mathematically, stochasticity means that the trajectories for each simulation are slightly different from one another and computationally intensive simulations are often required to follow the time evolution of the system dynamics. Our earlier studies [39], [40] have shown the potential of stochastic differential equations in the kinetics of signal transduction and ion channels. Mathematical analysis alone may be able to completely describe all the properties of interest in the case of simple random systems. However, mathematical analysis is not possible for more complex stochastic models, i.e. the complex stochastic models are analytically intractable.

The exact method to model chemical reactions, when diffusion is not taken into account, is the discrete-state chemical master equation (CME, [41]). However, the CME can rarely be solved and thus an algorithm called Gillespie stochastic simulation algorithm (SSA, [42], [43]) has been developed. The SSA presents an easy way to simulate the actual CME process and it is used more and more in computational modeling studies. In many cases, the SSA is slow to simulate and thus, we have chosen to introduce stochasticity into the reference model [31], [44] by the chemical Langevin equation (CLE, [45]), that is one type of stochastic differential equation. The CLE represents the continuous-state Markov model approximated from the exact CME. The CLE is much faster to simulate than the actual SSA when large volumes are considered but it can produce negative values when low concentrations are simulated [39]. However, for the system modeled in this study the CLE produce realistic results and can be thus used. When making the stochastic extension of the model, we need to assume volumes for the cytosol (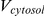) and ER (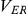) (see Table 1 for more information).

To describe the time-series behavior of the model, a set of equations (Equations 1–4) was introduced. 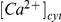, 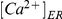, and 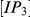 represent concentrations of cytosolic Ca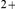, Ca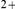 in the ER, and cytosolic 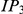, respectively. The fraction of active 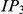 receptors on the ER membrane was termed 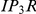. In the stochastic terms of Equations 1–4, 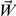 = 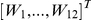 stands for the Brownian motion and 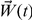 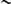 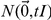. Furthermore, 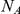 represents the Avogadro's number.
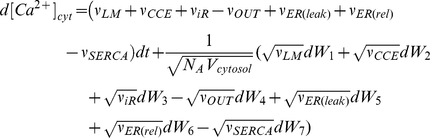
(1)

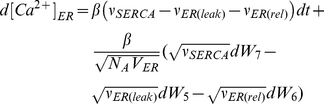
(2)

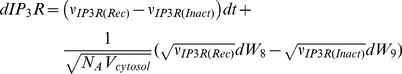
(3)

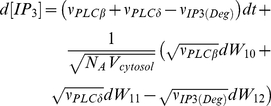
(4)


Due to the lack of fully understanding the phenomena related to CCE, the rate regulating capacitive Ca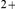 influx was assumed to be a nonlinear function of 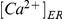, as described in [31]:
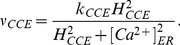
(5)


**Figure 3:**
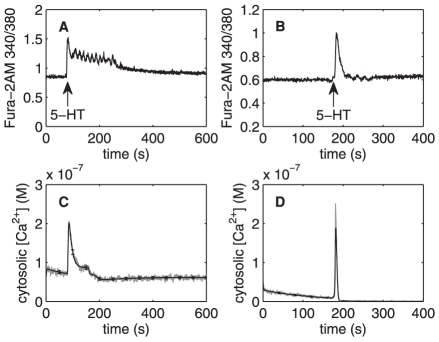
Changes in cytosolic Ca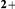 concentration induced by 5-HT (A–B) and computational simulations (C–D). **A.** Fast Ca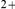 transient and a more sustained component are seen when 10 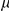
M 5-HT is added at t = 80 s. **B.** Changes in cytosolic Ca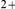 concentration induced by 1 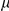
M 5-HT at t = 170 s in Ca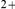 free media. Fast Ca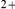 transient is seen but the more sustained component seen in Figure 2A is cut off. **C.** Model simulations of changes in cytosolic Ca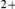 concentration induced by 10 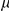
M input at t = 80 s. Fast Ca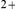 transient and a more sustained component are seen. **D.** Model simulations of changes in cytosolic Ca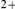 concentration induced by 1 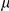
M input at t = 170 in simulated Ca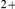 free media conditions: model rates 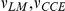, and 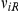 are set to zero. Fast Ca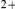 transient is seen but the more sustained component is cut off.

Earlier experimental results (cited in [31]) indicated that the transient component in cytosolic Ca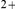 concentration was induced by the activation of the metabotropic receptor 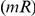 due to stimuli/input-evoked Ca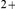 release from the intracellular stores, whereas the activation of the ionotropic receptor 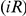 mediated the sustained component (similarly to our results; see Results and Figure 3A). In the reference model by Di Garbo et al. [31], ATP has an effect on 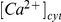 via both ionotropic and metabotropic receptors. The same is here assumed to 5-HT. Thus, the parts of the model (Equations 6 and 7) describing the ATP-induced Ca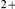 response in [31] is here used with some modifications to activate the model for astrocytic Ca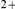 signaling with 5-HT and A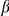.

In this study, the rate of Ca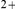 influx, induced by ionotropic receptors, from ECM to cytosol was modeled as in [31]:
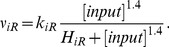
(6)


Similarly, the activation of G protein and PLC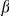 pathways, induced by metabotropic receptors, to promote the IP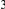 production were reformed from [31], [46] and modeled as:
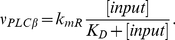
(7)


The remaining rate terms used in Equations 1–4 were taken from [46] and are explicitly formulated as Equations 8–16.
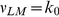
(8)

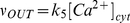
(9)

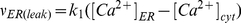
(10)

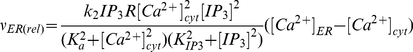
(11)

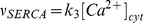
(12)

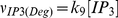
(13)

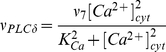
(14)

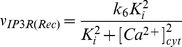
(15)

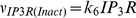
(16)


In addition, the following initial values were used: 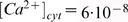 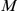, 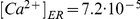 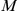, 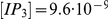 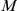, and 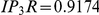.

## Results

To specifically study how the non-aggregated A
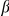
25–35 affects the metabotropic 5-HT receptor function, we added small amyloid peptide concentrations together with the transmitter and measured the ratio of emissions at 340 and 380 nm in Fura-2AM loaded rat cortical astrocytes in primary cultures. In some experiments L-glutamate was also added in aim to study the possible differences between glutamate and 5-HT receptor activation in these cells. The ratio of emissions is directly correlated to cytosolic Ca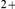 concentration 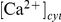. A deterministic model, introduced by Di Garbo et al. [31], was used as a reference model to which stochasticity was introduced by CLE in aim to reproduce the Ca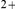 data measured with the used experimental conditions. Below we present the results obtained by combining the Fura-2AM measurements and computational simulations.

### Effects of 5-HT on the levels of cytosolic Ca
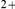


**Figure 4:**
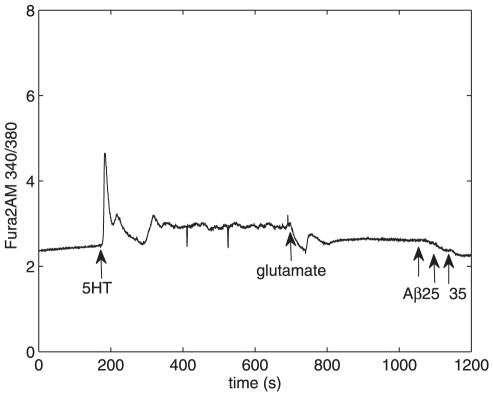
Changes in cytosolic Ca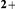 concentration induced by 5-HT, glutamate, and A
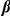
25–35 given at different times. Small change in cytosolic Ca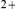 concentration is seen when 10 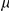
M 5-HT is added at t = 180 s. 50 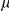
M glutamate reduces the 5-HT-induced enhancement of cytosolic Ca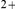 concentration at t = 700 s, whereas additions of 1 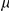
M A
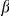
25–35 do not show an increment to cytosolic Ca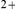 concentration at t = 1050, 1100, and 1130 s.

When the experiments were performed in solutions with normal external Ca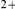, the addition of 5-HT every time induced a transient peak together with a more sustained increase in 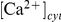 (Figures 3A and 4). When a lesser amount of 1 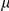
M 5-HT was added for 20 seconds in Ca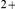 free medium, a single peak was seen, indicating release of Ca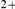 from intracellular stores (Figure 3B). The simulation of this is seen in Figure 3D. In Figures 3C and 3D, one realization of the chemical Langevin equation is printed in gray while the black traces represent the means and standard deviations for 1000 realizations. The lack of external Ca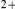 caused three of the components to be restrained in the six-component computational model, namely: 1) Ca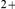 leak from extracellular matrix (ECM), 2) capacitive Ca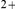 entry from ECM, and 3) Ca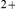 entry via ionotropic receptors. When rates 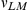, 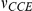, and 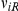 were set to zero the model simulations indeed produced fast transients without any sustained component. The model simulation closely resembled the experimental peak, except the peak duration was found to be shorter in simulations than in experiments (compare Figures 3B and 3D). With external Ca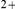 present, the simulation (illustrated in Figure 3C) shows a sustained component which, however, is shorter than seen in the experiments (compare Figures 3A and 3C).

### Effects of A
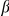
25–35 on the basal levels of cytosolic Ca
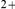


Our earlier studies [16] showed that only 36% of astrocytes responded to A
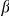
25–35 additions by transient increase in 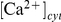, which returned back to baseline level after 1–4 minutes. In the present study, the mean value for the baseline 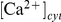 in control astrocytes was 2.39
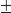
0.40 (mean in ratio 340/380 units 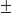 s.d.; n = 32), and 2.69
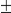
0.60 (n = 8) in those astrocytes which were similarly cultured and then incubated with 200 nM A
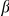
25–35 for 48 h (see the baseline at t = 0 … 180 s in Figure 2). There is no significant difference in the baseline values of the control and A
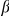
25–35-treated astrocytes (p = 0.098, which is 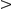
0.05; statistics were made using Anova module, Statistica, Statsoft Inc.), indicating that A
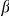
25–35 does not cause persistent change in the basal level of calcium in these cells.

### Synergistic effects of A
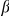
25–35 and transmitters on the levels of cytosolic Ca
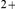


**Figure 5:**
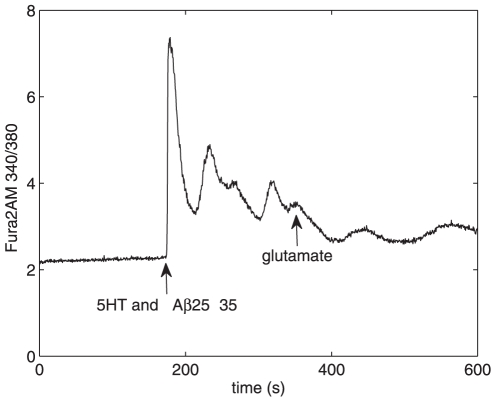
Synergistic effects of 5-HT and A
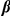
25–35 on cytosolic Ca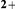 concentration. Substantial change in cytosolic Ca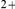 concentration due to synergistic effect of 10 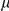
M 5-HT and 1 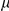
M A
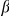
25–35 added at t = 180 s. 50 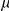
M glutamate, added at t = 350 s, reduces the enhancement of cytosolic Ca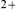 concentration.

The mean amplitude of 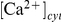 increase with simultaneous addition of 5-HT and A
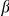
25–35 was statistically significantly different (p
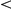
0.001) from the amplitude when 5-HT alone was added. 100% (n = 43) of studied astrocytes, with or without A
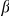
25–35 present, responded to 10 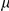
M 5-HT with a transient peak of increased 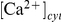. A
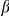
25–35 addition did not significantly change the mean duration or time constant of the first Ca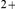 peak, but increased the peak amplitude, which reflects the magnitude of Ca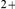 release from intracellular stores (compare traces in Figures 4 and 5). 1 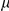
M A
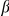
25–35, when added simultaneously with 10 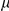
M 5-HT, caused a significant 163% increase in the mean Ca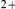 peak amplitude (n = 5) from the control value of 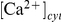 induced by 5-HT alone (n = 13). A lesser 75% increase was detected in cells incubated with 200 nM A
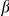
25–35 for 48 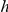 prior to adding 5-HT (n = 6, Figure 2). Astrocytes were also incubated with 10 nM A
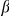
25–35, but the detected 5-HT-induced changes in 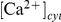 were then not significantly different from the control values.

**Figure 6:**
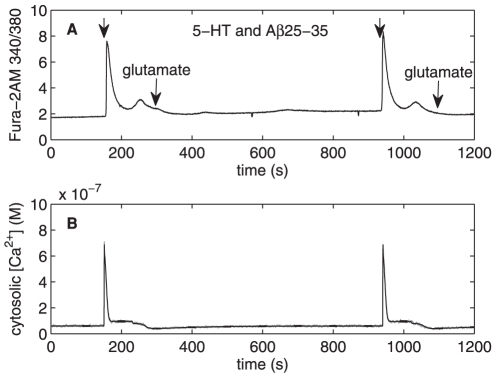
Effects of low stimulus frequency on cytosolic Ca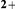 concentration; Fura-2AM measurements (A) and computational simulations (B). **A.** 1 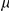
M A
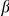
25–35 and 10 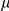
M 5-HT are added together at t = 150 s and t = 940 s. Glutamate is added at t = 300 s and t = 1100 s. The interval between the external stimuli is long enough to enable the intracellular Ca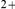 stores to fill up between the stimuli. Thus, the peak amplitude of the latter peak is not lower than the preceding one. **B.** Simulations of changes in cytosolic Ca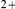 concentration induced by 10 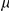
M external stimuli given at t = 150 s and t = 940 s. Model simulations reproduces the phenomena seen in Figure 6A.

Glutamate has earlier been shown to induce increase in intracellular Ca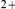 [17], and also in the present study 50 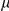
M glutamate induced increase in intracellular Ca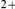 in 25% (n = 4) out of 16 astrocytes. Incubation of astrocytes with 10 nM or 200 nM A
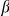
25–35 for 48 h increased the number of cells responding to glutamate to two cells out of four tested. When A
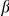
25–35 was added simultaneously with glutamate, 100% (n = 6) of astrocytes responded with a Ca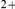 increase (data not shown). Furthermore, this study revealed another interesting interaction between intracellular Ca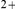 and glutamate: glutamate seems to be able to decrease 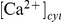, which has first been elevated by 5-HT, and to inhibit the Ca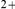-oscillations and return the Ca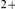 levels close to baseline (Figures 4, 5, and 6A). Glutamate may thus be able, by activating separate metabotropic receptors, to both increase 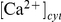 via release from intracellular stores and influx through L-type Ca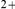 channels, and inhibit the Ca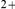 channel-mediated Ca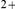 influx and oscillations. This phenomenon was seen in every cell tested (in 8 control astrocytes, 10 astrocytes incubated with 10 or 200 nM of A
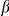
25–35, and 12 astrocytes where A
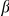
25–35 had been added simultaneously with glutamate). These effects of glutamate were not included in the computational model and synergistic effects of 5-HT and glutamate with A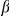 peptide fragments require further testing.

### The importance of intracellular Ca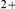 stores in Ca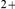 signaling

**Figure 7:**
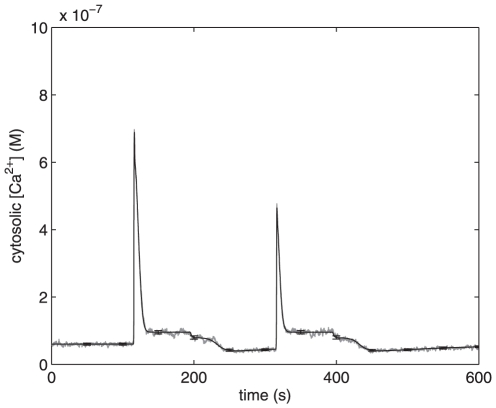
Simulated effects of high stimulus frequency on cytosolic Ca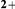 concentration. Model simulation of changes in cytosolic Ca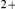 concentration induced by external stimuli. 10 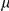
M stimuli, applied with a short interval at t = 115 s and t = 315 s, decrease the peak amplitude of the latter peak.

The ability of recurrent additions of the transmitter to induce a Ca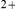 release from intracellular stores was tested using different frequencies of stimuli. If the stimuli (simultaneous addition of A
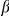
25–35 and 5-HT in experimental measurements) were given to the system more frequently, the peak amplitudes of the latter measurements were lower. This indicated the incomplete recovery from the desensitization of the receptor or the inadequate filling of the intracellular Ca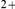 stores between the stimuli. However, the more sustained components, originating from Ca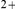 flux through plasma membrane, were similar, regardless of the frequency of stimuli. Simulations run with less/more frequent stimuli mimicked the experimental measurements (compare Figures 6A, 6B and 7). Thus, the Ca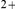 responses in simulations indeed depend on the preceding events. The more sustained component of Ca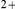 release seemed to remain both in the experimental results and simulations, regardless of the frequencies of the stimuli. In Figures 6B and 7, one realization of the chemical Langevin equation is printed in gray while the black traces represent the means and standard deviations for 1000 realizations.

## Discussion

One of the hallmarks of AD are the neuritic A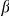 plaques. It is still an unresolved question how A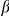 fragments start to form aggregates and at what concentrations they begin to affect the cellular interactions in the brain. We have here shown that even small amounts of A
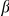
25–35 fragments in the rat cortical astrocytes can, together with 5-HT and glutamate, induce meaningful changes in the intracellular Ca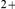 concentration. A
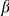
25–35 together with 5-HT caused an enhanced first peak of intracellular Ca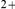 representing the release from intracellular stores mediated by 5-HT2A receptor. The glutamate induced increase in Ca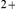 release from stores would most probably be mediated by a Group I (type 1 or 5) mGluR found in cortical astrocytes [17]. The observed additional inhibitory effect of glutamate could be the result of the activation of the group II metabotropic glutamate receptors which are known to reduce the voltage-sensitive Ca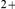 currents and be potential targets for neurological disorders (see [47], [48]).

In this study, we used data and a computational model to characterize the Ca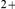 transients associated with synergistic effects of A
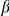
25–35 and transmitter 5-HT in rat cortical astrocytes. To our knowledge, this is the first such study. In the experimental part of this study, it was shown that 5-HT and A
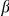
25–35, when added together, clearly increased the amplitudes of the Ca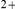 signals. Addition of A
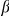
25–35, 5-HT, or glutamate alone was not able to induce that several-fold increment to the intracellular Ca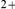, which was seen when A
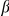
25–35 and 5-HT were added together. The abnormal increase in intracellular Ca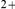 may in its turn trigger a complex cascade of a variety of molecular events in the intracellular signaling pathways [16], [17], [49]–[53]. The measured Ca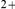 signals indicate the activation of 5-HT2A receptor followed by G protein, PLC, and IP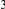 mediated Ca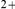 release from intracellular Ca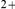 stores. An additional Ca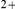 influx through voltage-sensitive and -insensitive Ca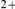 channels might be involved, as presented in [27]. Changes in astrocytic Ca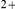 signaling are prone to cause widespread alterations in neuronal network function and can lead to neurological disorders (reviewed in [54]).

In the computational part of this study, a mathematical model by Di Carbo et al. [31], for simulating intracellular Ca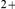 processes, was selected to be the basis for developing a more adequate model. Other models presenting Ca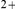 signaling in astrocytes (such as in [55]) include the flux of Ca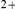 from/to ECM, pumping Ca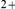 to ER, and Ca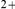 release from ER. The model selected for the present study includes six components which affect the intracellular Ca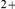 concentration: 1) Ca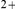 leak from/to ECM, 2) capacitive Ca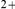 entry from ECM, 3) Ca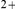 entry via ionotropic receptors, 4) Ca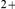 leak from intracellular stores, such as ER, 5) storage of Ca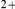 to ER via SERCA pumps, and 6) Ca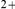 release from ER mediated by IP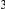. Due to different experimental setups, some of the components in the six-component model had to be restrained. The hypothesis about Ca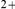 liberation from the intracellular stores was first experimentally verified, and then reproduced by simulations. The simulations supported the experimental findings in both Ca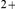 free media and with normal extracellular Ca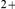 containing environment. The variability of biological signals cannot be accurately mimicked by deterministic models alone, which justified the use of stochastic methods.

A mathematical model, presented in this study, integrates data from several experimental sources and thus provides a way to computationally follow Ca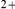 changes in biologically relevant conditions. Here, the stochastic model was able to reproduce the Ca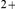 signals seen in the experimental Fura-2AM measurements. Potential pitfall of modeling, in general, is the inadequate experimental data. Experiments should originally be designed also to fulfill the demands of a modeling approach, including the need of considerable amount of repetitions, relevant statistics, and adequate metadata. When new components, describing cellular functions, will be added in the model, it will help to explore further the possible mechanisms behind the measured Ca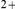 signals. This may advance the study of astrocytic Ca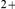 signals and their effects on neuronal networking in the central nervous system, by adding information of the intracellular targets activated by Ca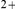 transients (studies on astrocytic Ca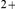 waves are reviewed in [56]). Calcium transients are known to affect the important intracellular Ca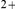 sensitive peptides, such as protein kinases and phosphatases. In addition, the passage of Ca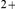 signals could lead to the priming of the astrocytes, thus modifying forthcoming astrocytic responses, setting the cellular basis for plasticity in glial cells [56]. Leissring et al. [57] have discussed the possibility that mutations in presenilin 1 (one of the factors in familial AD involved in the accumulation of amyloid 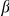 fragments in the brain) may change the activity of the ER Ca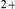-ATPases, e.g., SERCA. ATPases are associated with pumping the cytosolic Ca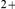 into the ER lumen, leading eventually to higher concentration of Ca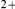 in ER. Amyloid 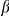 peptide accumulation may lead to higher-amplitude [Ca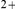]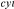 signals, have an effect on other Ca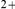-induced release, and increase intracellular IP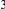 sensitivity [57]. Thus, the exceptional cytosolic Ca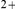 signals via ER, overfilled with Ca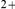, may explain the Ca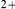 changes detected in the familial AD. Possible extension of the here developed stochastic model could be the incorporation of some specific IP
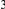
R model into the proposed model to study the role of altered IP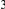 sensitivity on the overall Ca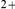 signaling.

The simulations run with our stochastic model did not take into account the possibility that the synergistic effects of A
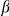
25–35 and 5-HT could be due to increased activation of, e.g., SERCA pumps. In addition, the pitfall of the here introduced stochastic model is that it does not take into account spontaneous Ca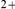 signaling in astrocytes (modeled, e.g., in [55]). To include these phenomena into our stochastic model would need further studies and tuning of model parameters. Progressive inclusion of additional components could lead to a still more realistic model of the Ca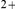 signaling in astrocytes. In general, a better understanding of the involvement of astrocytes in the developing pathology of Alzheimer's disease is of great importance for the future development of diagnosis and treatment. Early diagnosis of AD is important for initiating treatment and for understanding the pathobiology of the disease [58]. A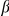-induced astrocyte activation is thought to have a critical role in the mechanisms of neurodegeneration in AD [59], as astrocytes signal to neurons in response to a physiological stimulus (see, e.g., [60]). The active participation of astrocytes in synaptic processes is of utmost importance for physiology of the nervous system [61], [62]. Studies combining experimental and computational experiments, like the present one, are required as they may provide us novel viewpoints and help explaining the possible mechanisms behind certain experimental findings.
